# Internal Limiting Membrane Flap Versus Conventional Peeling for Idiopathic Full Thickness Macular Holes: A Registry Analysis of 2990 Eyes

**DOI:** 10.1111/ceo.70010

**Published:** 2025-10-30

**Authors:** Zi Jin, Mohammad Amin Honardoost, Ee Lin Ong, Ahmad Reza Pourghaderi, Fred K. Chen, Weng Onn Chan, Prakshi Chopra, Mitchell Lee, Abhishek Sharma, Gurmit Uppal, Penelope J. Allen, Rohan W. Essex, Adrian T. Fung, Australian and New Zealand Society of Retinal Specialists Retinal Surgery Registry Group

**Affiliations:** ^1^ Queen Mary University of London UK; ^2^ Clinical Outcomes Data Reporting and Research Program, Department of Public Health and Preventative Medicine Monash University Victoria Australia; ^3^ Sydney & Sydney Eye Hospital New South Wales Australia; ^4^ Centre for Ophthalmology and Visual Science The University of Western Australia Perth Western Australia Australia; ^5^ Department of Ophthalmology Perth Children's Hospital Perth Western Australia Australia; ^6^ Centre for Eye Research Australia, Royal Victorian Eye and Ear Hospital East Melbourne Victoria Australia; ^7^ Vitreoretinal Unit Royal Victorian Eye and Ear Hospital East Melbourne Victoria Australia; ^8^ Department of Surgery (Ophthalmology) University of Melbourne East Melbourne Victoria Australia; ^9^ The University of Adelaide Adelaide Australia; ^10^ Department of Ophthalmology Royal Adelaide Hospital Adelaide Australia; ^11^ Sight Eye Institute Sydney Australia; ^12^ Department of Ophthalmology, Faculty of Medicine, Health and Human Sciences Macquarie University Hospital Sydney New South Wales Australia; ^13^ Department of Ophthalmology Westmead Hospital Sydney New South Wales Australia; ^14^ University of Queensland Australia; ^15^ Royal Brisbane & Women's Hospital Queensland Australia; ^16^ Moreton Eye Group Brisbane Queensland Australia; ^17^ Academic Unit of Ophthalmology, Medical School Australian National University Canberra Australia; ^18^ The Royal Victorian Eye and Ear Hospital Melbourne Australia; ^19^ Ophthalmology Department Canberra Hospital Garran Australian Capital Territory Australia; ^20^ Faculty of Medicine and Health The University of Sydney Sydney New South Wales Australia; ^21^ Zhongshan Ophthalmic Centre, Sun Yat‐Sen University Guangzhou China

**Keywords:** registries, retinal perforations, retrospective studies, vitrectomy

## Abstract

**Background:**

To compare the anatomical and functional outcomes of internal limiting membrane (ILM) flap and conventional ILM peeling in idiopathic full‐thickness macular holes (FTMHs).

**Methods:**

Retrospective cohort study of all eyes treated with vitrectomy and ILM peeling (ILM‐P) with or without ILM flap (ILM‐F) for primary idiopathic FTMH repair in the Australian and New Zealand Society of Retinal Specialists (ANZSRS) Registry between 2006 and 2023. Propensity score weighting and multivariable regression analysis adjusted for baseline characteristics and covariates, including surgeon grade, lens status, and follow‐up duration, were used to evaluate hole closure rate and best corrected visual acuity (BCVA) change at 3 months.

**Results:**

Two thousand nine hundred ninety eyes of 2905 patients were included (mean age 69 ± 9 years). One Hundred Ninety‐nine eyes underwent ILM‐F and 2871 underwent ILM‐P. On weighted multivariable regression analysis, ILM‐F showed higher odds of hole closure compared to ILM‐P (OR = 2.97, 95% CI: 1.08–8.20, *p* = 0.04). The adjusted closure rate was > 95% across all hole sizes in the ILM‐F group, while only falling below 90% for X‐large + holes (> 550 μm) in the ILM‐P group. No significant difference in BCVA gain was observed between the two groups at 3 months (*p* = 0.08). The effects of ILM‐F compared to ILM‐P were consistent across all hole sizes.

**Conclusions:**

Although the ILM‐F technique was more effective in idiopathic FTMH closure, visual acuity outcomes were comparable to conventional ILM peeling. These findings suggest that ILM‐F is not required for the treatment of small and medium FTMHs.

## 1 Introduction

Full thickness macular holes (FTMHs) are anatomical defects at the fovea, characterised by a disruption in the neural retinal layers [1]. They have a significant impact on vision, which may present as a loss of central vision, metamorphopsia, or scotomas. Idiopathic FTMHs are more common in the elderly and have an estimated incidence of 7.8 eyes per 100,000 individuals [2]. Surgical treatment of FTMHs involves pars plana vitrectomy, posterior hyaloid detachment if attached, and intravitreal gas tamponade. The internal limiting membrane peeling (ILM‐P) was introduced in 1997 by Eckardt et al. [3] and has become the mainstay approach for FTMHs, with up to 100% anatomical success [4, 5]. However, the challenge lies in large (> 400 μm) and chronic FTMHs where closure rates fall below 90% [4, 6, 7].

The inverted ILM flap (ILM‐F) was first described by Michalewska et al. [8] in 2009 to improve surgical outcomes in large FTMHs. In 50 eyes treated with ILM‐F, 98% closure was achieved [8]. Since its introduction, closure rates have reached 98.5% in large (> 400 μm) FTMHs [8, 9, 10]. Recent studies suggest that a higher threshold of approximately 500–650 μm may be more appropriate for the ILM‐F technique due to a lack of anatomical benefit in smaller holes and adverse effects on the outer retinal layers [4, 5, 11, 12]. Many of these studies primarily focus on anatomical outcomes rather than functional outcomes.

To address this knowledge gap, the anatomical and functional outcomes of the ILM‐F and conventional ILM‐P techniques for idiopathic FTMHs were evaluated within a large, bi‐national surgical registry dataset.

## 2 Methods

A retrospective analysis was performed on all patients from the Australian and New Zealand Society of Retinal Specialists Retinal Surgery (ANZSRS) Registry with idiopathic full thickness macular holes (FTMHs) who underwent vitrectomy surgery for primary macular hole repair between October 2006 and November 2023. The ANZSRS Registry is an online registry of vitreoretinal surgery performed by participating public and private operating centres in Australia and New Zealand. This study was approved by the Royal Australian and New Zealand College of Ophthalmologists Human Research Ethics Committee (Study 101.19 e A Retinal Surgery Logbook and Audit Tool) and adhered to the tenets of the Helsinki Declaration and the Good Medical Practice Guidelines.

Eyes were included if the FTMH was repaired using ILM‐F (inversion of ILM into or over the hole) or conventional ILM‐P (complete removal of ILM around the hole). As this was a registry‐based study, the exact ILM‐F technique performed was not specified and was at the discretion of the surgeon. Exclusion criteria included: FTMHs secondary to trauma, myopia, or any other ocular pathologies; recurrent or persistent FTMHs; other procedures to close the MH; a follow‐up duration of less than 3 months; and inadequate baseline or follow‐up documentation. In cases of bilateral FTMH repairs, both eyes were included if they met the inclusion and exclusion criteria.

Baseline characteristics included patient demographics, best corrected visual acuity (BCVA) measured with a Snellen chart and converted to logarithm of the minimum angle of resolution (logMAR) for statistical analysis, laterality, FTMH size, patient‐reported duration of symptoms, lens status, and whether vitreous was attached to FTMH margins. FTMHs were classified as small (≤ 250 μm), medium (> 250 and ≤ 400 μm), or large (> 400 and ≤ 550 μm) as described by the International Vitreomacular Traction Study (IVTS) [13], and X‐large (> 550 and ≤ 800 μm), XX‐large (> 800 and ≤ 1000 μm) or giant (> 1000 μm) based on the Classification for Large Macular Hole Studies (CLOSE study group) [14]. Surgical information included surgeon grade (consultant/trainee), gauge of surgical instrumentation, posterior vitreous detachment induction, ILM stain, combined phacoemulsification/vitrectomy surgery, type of tamponade agent, and duration of prescribed face‐down posturing. At the final follow‐up, time (in days post‐operatively), BCVA, macular hole closure, and lens status were also recorded.

### 2.1 Outcomes

The primary endpoint of this study was the rate of hole closure on optical coherence tomography (OCT) scans after a single procedure at the follow‐up visit. The secondary endpoint was BCVA change (∆ logMAR) calculated by the difference between pre‐operative BCVA and post‐operative BCVA at the 3‐month follow‐up.

### 2.2 Statistical Analysis

All statistical analyses were carried out using statistical software R (version 4.2.2, R Core Team, 2021) [15]. Due to limited sample sizes in the small, XX‐large, and giant groups, the MH size groupings were consolidated into three categories: small/medium, large, and X‐large + (including XL, XXL, and giant). Categorical variables were summarised as frequencies and proportions, while continuous variables were presented as either median (Me) and interquartile range (Q1, Q3) or mean and standard deviation (SD). For categorical data, Chi‐square (χ2) test and Fisher's exact test were applied based on sample size. For continuous data, independent t‐test and Wilcoxon rank‐sum test differences were used depending on the normality of the data. A *p* value of < 0.001 indicates non‐normality using the Shapiro–Wilk test.

Univariable regression analysis was first conducted to identify covariates significantly associated with the outcome, including surgical technique, age, gender, pre‐operative BCVA, MH size, duration of MH, lens status (pre‐ and post‐operative), vitreous attachment to hole margins, duration of follow‐up, and grade of the surgeon. Significant covariates (*p* < 0.05) were included in the stratified overlap‐weight propensity adjustment and weighted multivariable regression analysis. To account for covariate imbalance and potential residual confounding, first overlap‐weight propensity‐score adjustment (estimand = average treatment effect in the overlap population) within each hole‐size stratum was applied using the WeightIt package. Covariate balance before and after weighting was evaluated with standardised mean differences, targeting |SMD| < 0.10 as the balance criterion. Stratum‐specific weights were combined, and a weighted multivariable regression model was fit using the svyglm function with a binomial or gaussian error distribution for binary and continuous outcomes, respectively. In all multivariable regression analyses, interaction terms between hole size groupings and closure technique were assessed and included in the final model where they were statistically significant. Overlap‐weighted estimated marginal means (EMMs) were calculated and reported. In all regression analyses, only complete cases (i.e., cases with valid values for both the outcome variables and all covariates) were included. A *p* value of < 0.05 was considered statistically significant.

## 3 Results

### 3.1 Clinical Profile

**FIGURE 1:**
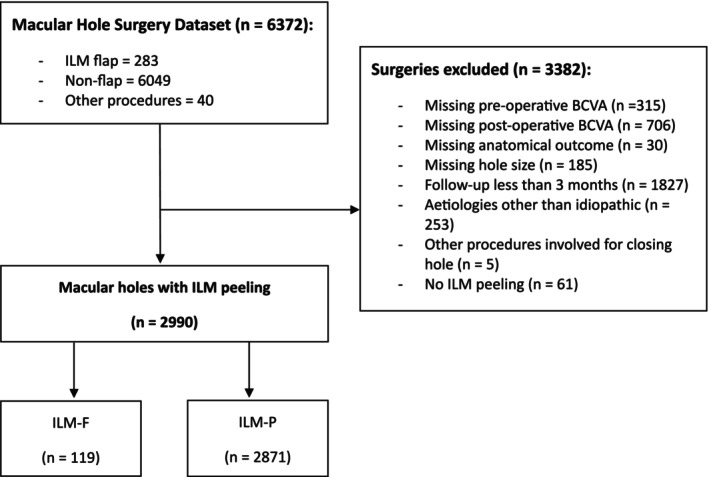
Flowchart illustrating identification of patients included from the ANZSRS registry macular hole surgery dataset. Abbreviations: BCVA, best corrected visual acuity, ILM, internal limiting membrane; ILM‐F, internal limiting membrane flap; ILM‐P, internal limiting membrane peeling.

Two thousand nine hundred ninety eyes from 2905 patients were identified from the Macular Hole Surgery dataset of the ANZSRS registry, 119 eyes underwent ILM‐F and 2871 eyes underwent conventional ILM‐P (Figure 1). The mean age of the population was 69 ± 9 years. 2019 (67.5%) eyes were from female patients. The median MH size was 300 μm (interquartile range [IQR], 200–422 μm) and the median follow‐up time was 112 days (IQR, 84–153 days). Bilateral FTMHs were present in 85 patients.

Table 1 summarizes the baseline characteristics of the ILM‐F and ILM‐P groups. There were no statistically significant differences between the two groups in terms of sex, pre‐operative vitreous attachment to hole margins, and pre‐operative lens status. The mean age was significantly higher in the ILM‐F group (71 ± 7 years) compared to the ILM‐P group (69 ± 9 years, *p* = 0.013). The median pre‐operative MH size was significantly greater in the ILM‐F group (521 μm) compared to the ILM‐P group (296 μm, *p* < 0.001). The distribution of MH size groupings differed between the two groups, with a greater proportion of MHs > 400 μm in the ILM‐F group (80.6%) compared to the ILM‐P group (25.6%, *p* < 0.001). The MHs in the ILM‐F group had a longer median symptom duration (6 vs. 3 months, *p* < 0.001) and a worse median pre‐operative BCVA (0.9 logMAR vs. 0.7 logMAR, *p* < 0.001) compared to those in the ILM‐P group. In contrast, the ILM‐P group had a higher consultant‐to‐trainee ratio (8.2:1) compared to the ILM‐F group (3.1:1; *p* < 0.001). Post‐operatively, the proportion of aphakic/pseudophakic eyes was significantly higher in the ILM‐F group (64.7%) compared to the ILM‐P group (48.6%).

**TABLE 1:** Baseline characteristics of patients undergoing primary idiopathic full thickness macular hole repair with ILM Flap or conventional ILM peeling technique.

Baseline characteristic	ILM‐F *n* = 119	ILM‐P *n* = 2871	*p*
Age, mean (SD), years	71 (7)	69 (9)	0.013
Female sex, *n* (%)	82 (68.9%)	1937 (67.5%)	0.7
Pre‐operative MH size, Me (Q1, Q3), μm	521 (428650)	296 (200408)	< 0.001
Pre‐operative MHs classified into size groupings, *n* (%)			< 0.001
Group 1			
Small (≤ 250 μm)	1 (0.8%)	1094 (38.1%)	
Medium (> 250 and ≤ 400 μm)	22 (18.5%)	1042 (36.3%)	
Group 2			
Large (> 400 and ≤ 550 μm)	41 (34.5%)	450 (15.7%)	
Group 3			
X‐Large (> 551 and ≤ 800 μm)	48 (40.3%)	230 (8.0%)	
XX‐Large (> 801 and ≤ 999 μm)	6 (5.0%)	35 (1.2%)	
Giant (≥ 1000 μm)	1 (0.8%)	20 (0.7%)	
Duration of hole, Me (Q1, Q3), months	6 (4.12)	3 (2.6)	< 0.001
Pre‐operative vitreous attached to hole margins, *n* (%)	49 (42.6%)	1390 (50.4%)	0.10
Pre‐operative lens status, *n* (%)			0.082
Phakic	85 (71.4%)	2238 (78.2%)	
Aphakic/Pseudophakic	34 (28.6%)	625 (21.8%)	
Baseline BCVA, Me (Q1, Q3), logMAR	0.9 (0.7, 1.0)	0.7 (0.5, 0.9)	< 0.001
Surgeon grade, *n* (%)			< 0.001
Consultant	90 (75.6%)	2494 (89.1%)	
Fellow/Registrar	29 (24.4%)	304 (10.9%)	

Abbreviations: BCVA, best corrected visual acuity; ILM‐F, internal limiting membrane flap; ILM‐P, internal limiting membrane peeling; Me, median; MH, (full thickness) macular hole; Q1, lower quartile; Q3, upper quartile.; SD, standard deviation.

### 3.2 Anatomical Outcomes

**FIGURE 2:**
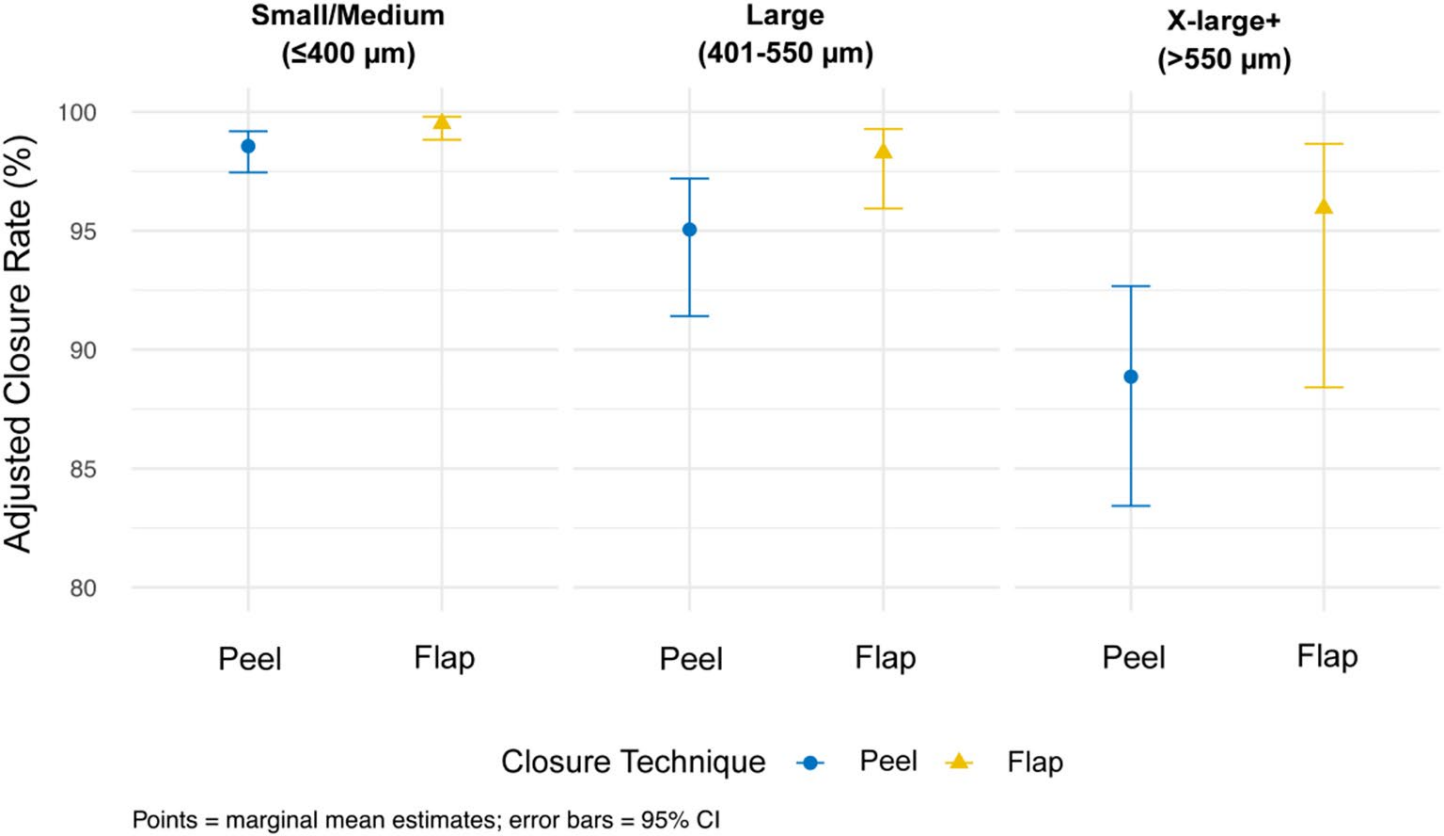
Estimated marginal means of 3‐month hole closure rate for ILM flap and ILM peel techniques across macular hole sizes.

Unadjusted MH closure was achieved in 115/119 cases (96.6%) in the ILM‐F group and 2778/2871 cases (96.8%) in the ILM‐P group. In the univariable analysis, significant variables were namely pre‐operative vitreous attachment to hole margins, MH size, duration of MH, baseline BCVA, and surgeon grade (Table 2). Closure rates following adjustment for covariables were 98.5% in the ILM‐F group and 95.6% in the ILM‐P group. Weighted multivariable regression analysis showed that ILM‐F was significantly associated with higher odds of hole closure compared to ILM‐P (OR = 2.969, 95% CI: 1.075 to 8.199, *p* = 0.036). No significant interaction effects were observed, indicating that the odds of anatomical success with ILM‐F and ILM‐P did not significantly differ by MH size. Among the independent predictors of MH closure, pre‐operative vitreous attachment to the hole margins significantly increased the odds of closure (OR = 2.632, 95% CI: 1.382 to 5.014, *p* = 0.003). Factors associated with reduced odds of anatomical success were MH size groupings > 400 μm (X‐large + OR = 0.117, 95% CI: 0.061 to 0.226, *p* < 0.001; large OR = 0.282, 95% CI: 0.151 to 0.525, *p* < 0.001) and surgeries performed by fellows/registrars (OR = 0.451, 95% CI: 0.205 to 0.993, *p* = 0.048). Figure 2 illustrates a reduction in the adjusted closure rates with increasing MH size for both ILM‐F and ILM‐P techniques. Closure rates dropped below 95% for X‐large+ MHs in the ILM‐P group, while rates of ILM‐F remained consistently above 95% across all size groupings.

**TABLE 2:** Univariable and weighted multivariable regression analysis for hole closure rate (reference: failed to close).

Variables	Univariable regression			Multivariable regression		
	OR	95% CI	*p*	OR	95% CI	*p*
Technique (baseline = ILM‐P), ILM‐F	0.962	0.394, 3.185	> 0.9	2.969	1.075, 8.199	0.036
Significant variables in multivariable regression						
Pre‐operative vitreous attached to hole margins (baseline = no), Yes	1.928	1.265, 2.992	0.003	2.632	1.382, 5.014	0.003
Surgeon grade (baseline = consultant), Fellow/registrar	0.423	0.263, 0.705	< 0.001	0.451	0.205, 0.993	0.048
MH size (baseline = small/medium), Large	0.295	0.176, 0.499	< 0.001	0.282	0.151, 0.525	< 0.001
MH size (baseline = small/medium), X‐Large+	0.139	0.086, 0.225	< 0.001	0.117	0.061, 0.226	< 0.001
Non‐significant variables in multivariable regression						
Age (in years)	0.981	0.957, 1.005	0.14	—	—	—
Gender (baseline = female), Male	1.256	0.810, 2.003	0.3	—	—	—
Pre‐operative duration of hole (in months)	0.979	0.968, 0.990	< 0.001	0.988	0.972, 1.003	0.12
Pre‐operative lens status (baseline = phakic), Aphakic/pseudophakic	0.966	0.606, 1.602	0.9	—	—	—
Post‐operative lens status (baseline = phakic), Aphakic/pseudophakic	1.107	0.733, 1.675	0.6	—	—	—
Baseline BCVA	0.280	0.161, 0.497	< 0.001	0.747	0.286, 1.950	0.6
Follow‐up duration (in days)	1.001	1.000, 1.003	0.3	—	—	—

Abbreviations: BCVA, best corrected visual acuity; CI, confidence interval; Large, > 400 and ≤ 550 μm; Medium, > 250 and ≤ 400 μm; MH, macular hole; OR, odds ratio; Small, ≤ 250 μm; X‐Large + (includes X‐Large > 550 and ≤ 800 μm, XX‐Large > 800 and ≤ 1000 μm, and Giant, > 1000 μm).

### 3.3 Functional Outcomes

**FIGURE 3:**
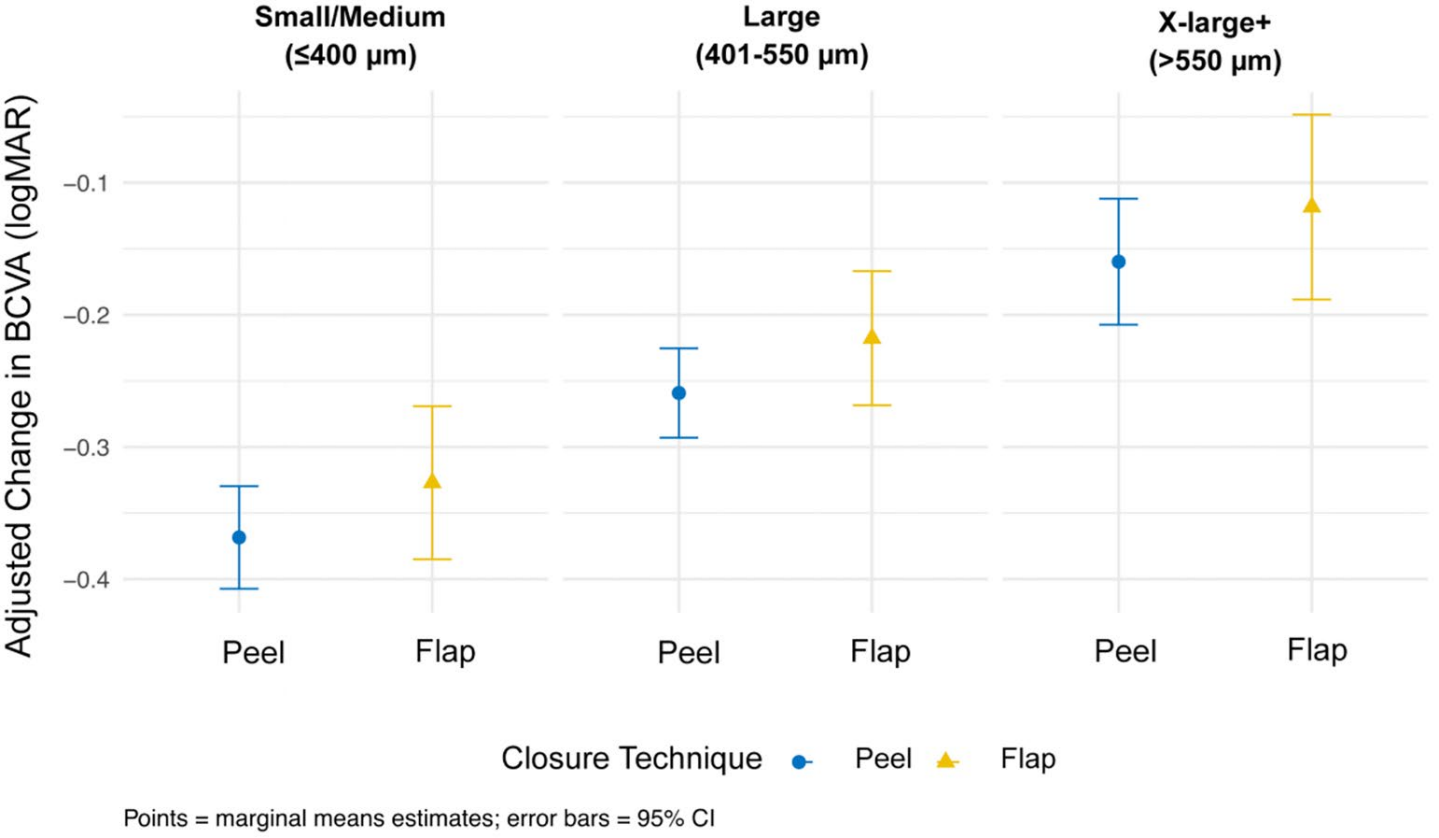
Estimated marginal means of 3‐month visual acuity change for ILM flap and ILM Peel techniques across macular hole sizes.

The adjusted gain in BCVA from baseline to 3‐month follow‐up was −0.221 logMAR in the ILM‐F group and −0.262 logMAR in the ILM‐P group. In the univariable analysis, baseline BCVA, MH size, duration of MH, pre‐operative and post‐operative lens status, and follow‐up duration were found to be significantly associated with BCVA change (Table 3). After adjusting for these covariates, there was no significant difference in BCVA change between the two closure techniques (*β* = 0.041, 95% CI: −0.005 to 0.088, *p* = 0.081). Figure 3 illustrates the adjusted visual improvement with both ILM‐F and ILM‐P techniques across all MH size groupings. No significant interaction effects suggest that comparable visual outcomes between ILM‐F and ILM‐P were consistent across all MH sizes. Two predictors of greater BCVA gain were worse baseline BCVA (*β* = −0.659, 95% CI: −0.721 to −0.597, *p* < 0.001) and post‐operative aphakic/pseudophakic lens status (*β* = −0.144, 95% CI: −0.201 to −0.087, *p* < 0.001). Both large (*β* = 0.109, 95% CI: 0.058 to 0.161, *p* < 0.001) and X‐large + (*β* = 0.209, 95% CI: 0.145 to 0.272, *p* < 0.001) MHs were associated with less BCVA gain at 3 months. Pre‐operative aphakic/pseudophakic lens status was also significantly associated with less BCVA gain (*β* = 0.061, 95% CI: 0.006 to 0.117, *p* = 0.031).

**TABLE 3:** Univariable and weighted multivariable regression analysis for 3‐month change in BCVA (ΔlogMAR).

Variables	Univariable regression			Multivariable regression		
	Beta	95% CI	*p*	Beta	95% CI	*p*
Technique (baseline = ILM‐P), ILM‐F	−0.025	−0.079, 0.029	0.4	0.041	−0.005, 0.088	0.081
Significant variables in multivariable regression						
Baseline BCVA	−0.619	−0.652, −0.587	< 0.001	−0.659	−0.721, −0.597	< 0.001
Post‐operative lens status (baseline = phakic), Aphakic/pseudophakic	−0.128	−0.148, −0.108	< 0.001	−0.144	−0.201, −0.087	< 0.001
Pre‐operative lens status (baseline = phakic), Aphakic/pseudophakic	−0.030	−0.054, −0.007	0.012	0.061	0.006, 0.117	0.031
MH size (baseline = small/medium), Large	−0.048	−0.078, −0.017	0.002	0.109	0.058, 0.161	< 0.001
MH size (baseline = small/medium), X‐large+	−0.009	−0.053, 0.035	0.7	0.209	0.145, 0.272	< 0.001
Non‐significant variables in multivariable regression						
Age (in years)	0.000	−0.001, 0.001	0.5	—	—	—
Gender (baseline = female), Male	0.006	−0.015, 0.027	0.6	—	—	—
Pre‐operative duration of hole (in months)	0.002	0.000, 0.004	0.049	0.003	0.000, 0.006	0.054
Pre‐operative vitreous attached to hole margins (baseline = no), Yes	−0.004	−0.024, 0.017	0.7	—	—	—
Follow‐up duration (in days)	0.000	0.000, 0.000	< 0.001	0.000	0.000, 0.000	0.6
Surgeon grade (baseline = consultant), Fellow/registrar	0.019	−0.016, 0.055	0.3	—	—	—

Abbreviations: BCVA, best corrected visual acuity; CI, confidence interval; Large, > 400 and ≤ 550 μm; Medium, > 250 and ≤ 400 μm; MH, macular hole; Small, ≤ 250 μm; X‐Large + (includes X‐Large > 550 and ≤ 800 μm, XX‐Large > 800 and ≤ 1000 μm, and Giant, > 1000 μm); β, beta‐coefficient.

## 4 Discussion

Using binational registry data from a cohort of 2990 eyes, our results showed that the ILM flap technique has an anatomical benefit in the primary closure of idiopathic FTMHs with an OR of 2.97. Visual outcomes were no different using ILM‐F and conventional ILM‐P.

There is ongoing debate on whether ILM flaps yield anatomical and functional benefits over conventional complete ILM peeling and whether there is a size cut‐off at which FTMHs are most likely to benefit. Prior studies assessing this show conflicting results and are presented in Table 4. Limitations of many of these studies include small sample sizes, non‐randomisation and non‐comparative analyses, inclusion of non‐idiopathic macular holes, heterogeneity in follow‐up duration, different surgical techniques, different definition of hole closure [10, 17], lack of reporting of functional outcomes and poor adjustment for baseline characteristics including macular hole size, duration and lens status.

**TABLE 4:** Studies included in literature review for large and all‐sized macular holes.

Year	Author	Study type	MH size (μm)	ILM peel, *N*	ILM flap, *N* (technique)	Follow‐up (months)	Closure rate	Visual acuity
2018	Kannan [16]	RCT	600–1500	30	30 (classic)	6	*No significant difference*	*No significant difference in absolute VA and VA gain*
2018	Manasa [17]	RCT	> 600	48	43 (inverted cover)	3	*No significant difference*	*ILM flap better absolute VA (0.67) than ILM peel (0.86), p = 0.001* *Only ILM Flap had a significant VA improvement*
2018	Velez‐Montoya [18]	RCT	> 400	12	12 (classic), 14 (free)	3	*No significant difference*	*No across‐group comparison (only ILM flap had significant VA improvement from baseline, p < 0.007)*
2010	Michalewska [8]	RCT	> 400	51	50 (classic)	12	*ILM peel (88%) vs. ILM flap (98%), no p value*	*ILM flap better absolute VA (0.17 vs 0.28, p = 0.001)*
2025	Jin	Retrospective cohort	Mean: 300 μm (IQR: 200 to 422)	2871	119	3	*ILM flap higher closure than ILM Peel (OR = 2.97, 95% CI: 1.08–8.20, p = 0.04)*	*No significant difference*
2024	Bencheqroun [19]	Retrospective cohort	> 650	58	16 (classic)	10	*No significant difference*	*ILM peel better VA gain (0.62 vs. 0.39, p = 0.04)* *No significant difference in absolute VA*
2024	Macchi [20]	Retrospective cohort	> 500	59	18 (classic), 36 (pedicle transposition), 16 (free)	6	*ILM flaps (97%, 100%, 100%) higher closure than ILM peel (59%), p < 0.0001* *No significant difference between flaps, p = 0.6*	*No significant difference except pedicle transposition flap superior to free flap (ETDRS letters +27 vs. +12, p = 0.02)*
2024	Riding [11]	Retrospective cohort (BEAVRS)	> 500	110	80 (superior)	Min. 2	*ILM flap higher closure (96.3%) than ILM Peel 94 (85.5%), p = 0.023*	*No significant difference in absolute VA, VA gain, and VA success*
2024	Chen [12]	Retrospective cohort	400–650	37	32 (temporal)	6	*No significant difference*	*ILM peel better absolute VA (0.36 vs. 0.54, p = 0.006)*
2024	Suarez [21]	Retrospective cohort	38–1109	130	30 (inverted—‘cover’)	Min. 3	*No significant difference*	*No significant difference*
2024	Morikawa [22]	Retrospective cohort	Mean: ILM flap (415 ± 173) vs. ILM peel (396 ± 201)	41	25 (classic)	12	*No significant difference stated*	*No significant difference in absolute VA*
2024	Ďurana [23]	Retrospective cohort	46–724	43	24 (inverted ILM flap without extra manipulation)	2	*No significant difference*	*No significant difference in absolute VA and VA gain*
2023	Koçak [10]	Retrospective cohort	> 600	32	28 (temporal)	6	*ILM flap (96.4%) higher closure than ILM peel (75.0%), p = 0.029*	*ILM flap better VA gain (0.61) than ILM peel (0.28), p = 0.001* *ILM flap better absolute VA (0.49) than ILM peel (0.70), p = 0.01*
2023	Dera [24]	Retrospective cohort	> 400	52	65 (classic and temporal)	14	*ILM flap (90.8%) higher closure than ILM peel (59.6%), p < 0.001*	*No significant difference*
2023	Chen [25]	Retrospective cohort	Mean: 519.46	34	15 (classic)	12	*No significant difference*	*No significant difference in absolute VA at 1 and 12 months*
2023	Michalewicz [26]	Retrospective cohort	ILM flap (421.90 ± 158.53) vs. ILM Peel (322.61 ± 159.62)	61	48 (classic)	Max. 6.4 years	*ILM flap (97.9%) higher closure than ILM peel (80.3%), p = 0.006*	*No significant difference in VA gain*
2023	Kwak [27]	Retrospective cohort	Mean: ILM Flap (393.69) vs. ILM Peel (385.73)	26	26 (superior)	12	*No significant difference*	*ILM flap better absolute VA (P < 0.05) and VA gain (p = 0.038) at 1 month* *No significant difference in VA gain at 3 months*
2023	Carballés [28]	Retrospective cohort	183–760	36	44 (superior)	6	*No significant difference*	*ILM flap better absolute VA (0.37) than ILM peel (0.51) at 3 months, p = 0.0125* *ILM flap better VA gain at 3 months, p = 0.0001* *No significant difference in absolute VA at 6 months* *ILM flap better VA gain at 6 months, p = 0.0126*
2021	Yan [29]	Retrospective cohort	154–1066	19	29 (inverted—‘cover’)	6	*No significant difference*	*No significant difference in absolute VA*
2020	Baumann [9]	Retrospective cohort	> 400	49	68 (classic)	12	*ILM flap (98.5%) higher closure than ILM peel (87.8%), p = 0.02*	*No significant difference in VA gain*
2019	Avci [30]	Retrospective cohort	> 400	18	15 (temporal)	12	*ILM flap higher closure (100%) than ILM peel (72.2%), p = 0.036*	*ILM flap better absolute VA (0.25) than ILM peel (0.56), p = 0.015* *ILM flap greater VA gain (0.91) compared to ILM peel (0.49 ± 0.59) at 12 months, p = 0.037*
2018	Yamashita [31]	Retrospective cohort	> 400	105	60 (classic)	Min. 6	*No significant difference*	*No significant difference in VA gain and VA success*
2018	Rizzo [32]	Retrospective cohort	Not stated	300	320 (classic)	9	*ILM flap (91.93%) higher closure than ILM peel (78.75%), p = 0.001*	*ILM flap better absolute VA (0.43 ± 0.31) than ILM peel (0.52 ± 0.42), p = 0.003*
2025	Tzoumas [33]	Systematic Review	Mainly large MHs (IQR 450–744 μm)	13 RCTs, 792 eyes	ILM flap (509–315, 61.9% ‘classic’ vs. 194, 38.1% ‘cover’ inverted), 283 ILM peel	6	*Adjusted odds ratio (OR) for ILM flap vs ILM peel 4.80 (95% CI, 2.77–8.30; p < 0.001)*	*ILM flap better VA improvement than ILM peel, mean difference of—0.14 (−0.18 to −0.09, p < 0.001)*
2024	Zhang [34]	Systematic Review	227.0–803.3	68 papers incl. 19 RCTs, 7257 eyes	ILM peel (6112), inverted ILM flap (1145)	—	*After sensitivity analysis with 3771 patients, 26 studies: ILM Flap (628.3m) larger than ILM Peel (452.2m) at baseline, p < 0.001* *ILM peel: very 100‐μm increment relatively lower closure rate (4.0%, 95% CI 1.4%–6.7%, p = 0.03)* *No significant association in ILM flap*	*Not studied*
2023	Rezende (CLOSE study) [14]	Systematic Review	> 400	31 papers, 1135 eyes	ILM peel (683), ILM flap (233), macular hydrodissection (64), human amniotic mem‐ brane graft (59), and autologous retinal transplantation (96)	3 years	*Did not compare groups*. *ILM peel best closure when FTMH 400–535 μm (96.8%); ILM flaps best closure when FTMH 400–799 μm (99.0%)*	*Did not compare groups*. *ILM peel best adjusted mean VA when FTMH 400–535 μm; ILM flap best adjusted mean VA when FTMH 400–799 μm*
2023	Ghoraba [35]	Systematic Review	> 400	4 RCTs, 285 eyes	ILM peel, classic flap	Min. 3	*ILM flap increases MH closure (risk ratio [RR] 1.10, 95% CI 1.02 to 1.18; p = 0.01)*	*No significant difference at 1 month* *ILM flap higher VA than ILM Peel at 3 months (MD−0.17 logMAR, 95% CI −0.23 to −0.10; p < 0.001)*
2020	Shen [36]	Systematic Review	> 400	4 RCTs and 4 case series, 593 eyes	ILM peel (325), inverted ILM flap (268)	Min. 3	*ILM flap higher closure than ILM peel (OR) = 3.95, 95% CI = 1.89 to 8.27; p = 0.0003*	*ILM flap VA change better than ILM Peel at 3 months (mean difference (MD) = −0.16, 95% CI = −0.23 to 0.09; p < 0.00001)* *No difference in VA change at 6 months*

*Note:* ‘Classic’ refers to the original inverted ILM flap technique by Michalewska et al. [8]; Studies demonstrating a statistically significant benefit of ILM‐F are shown in italics, while those demonstrating a statistically significant benefit of ILM‐P are shown with underlining.

Abbreviations: CI, confidence interval; ILM, internal limiting membrane; MH, macular hole; OR, odds ratio; RCT, randomised controlled trials; VA, visual acuity.

Michalewska et al. first described a 98% closure rate with the inverted ILM flap technique compared to 88% with conventional ILM peeling for large (> 400 μm) macular holes in a randomised controlled trial (RCT) [8]. Six retrospective cohort studies focusing on large idiopathic FTMHs showed that the ILM‐F technique achieved higher closure rates compared to the ILM‐P technique (Table 4) [9, 10, 11, 20, 24, 30]. In two systematic reviews, Ghoraba et al. [35] and Shen et al. [36] found that the ILM‐F increased anatomical closure by a relative risk of 10% and an OR of 3.95, respectively. Another recent systematic review by Tzoumas et al. [33], including 13 RCTs and 792 eyes of all MH sizes, showed a similar adjusted OR of 4.80. The authors found that ILM‐F was more likely beneficial for holes > 500 μm (OR = 3.14 to 9.64, *p* < 0.001). In this study, a hole closure rate of above 95% was achieved using the ILM‐F technique, which is considered a benchmark of success with MH repair.

The proposed mechanism of benefit for the inverted ILM‐F is its potential to act as a scaffold to promote the proliferation and migration of Müller cells and gliosis [8, 37, 38]. Using experimental monkey models, Shiode et al. [39] observed neuronal modelling from activated Müller cells produced neurotrophic factors and basic fibroblast growth factors on the ILM surface, only 10 days after ILM flap surgery. In addition, the ILM‐F is thought to function as a barrier to fluid entry from the vitreous into the subretinal space [39, 40].

Despite higher closure rates with the ILM‐F technique, our study did not find a functional benefit of the ILM‐F compared to conventional peeling. However, the follow up time was relatively short and many more eyes in the ILM‐F group had larger or more chronic holes that may take longer to recover full visual acuity. Greater BCVA gain was found in eyes with worse baseline BCVA. This likely reflects a ceiling effect in those with better baseline BCVA. The benefit of post‐operative aphakic/pseudophakic lens status probably reflects the impact of cataract surgery improving vision.

One RCT and 7 retrospective studies have found comparable visual outcomes between ILM‐F and ILM‐P [16, 21, 22, 23, 25, 29, 31]. Five retrospective studies corroborate our study findings where despite an anatomical superiority of ILM‐F, functional outcomes were no different [9, 11, 20, 24, 26]. In these cases, there was no difference in the recovery of the outer retinal layers [9, 10]. Two studies even showed that ILM‐P had a favourable visual acuity benefit over ILM‐F for idiopathic FTMHs. Bencheqroun et al. [19] conducted a retrospective study, which found that conventional ILM‐P was superior in BCVA gain compared to ILM‐F (*p* = 0.04) at a mean of 10 months. Chen et al. [12] found a better post‐operative BCVA at 1, 3, and 6 months (*p* = 0.039, 0.005, and 0.006) with ILM‐P [12]. They hypothesized that an ILM‐F might fill the hole after air‐gas exchange and impede external limiting membrane (ELM) and ellipsoid zone (EZ) recovery. Excessive gliosis could have cytotoxic effects on retinal neurons, leading to a worse visual prognosis [39]. Earlier studies identified this as foveal hyperreflective lesions, which were associated with EZ disruption [8, 39]. Iatrogenic trauma can also occur with insertion of ILM into the fovea [41, 42]. Larger FTMHs often favour ILM flap, where photoreceptor loss or atrophy may result in limited visual improvement.

Conversely, three studies showed that ILM‐F achieved a better post‐operative absolute or gain in visual acuity in larger MHs [10, 17, 30]. Recent studies suggest that the ILM‐F technique may offer superior early visual recovery compared to conventional ILM peeling, but longer‐term visual outcomes appear comparable between the two techniques. Carballés et al. [28] noticed a quicker recovery in visual acuity at 3 months using ILM‐F, but the difference in absolute BCVA was not significant at 6 months. Kwak et al. [27] observed an early recovery at 1 month, but no significant difference between the two groups at 3 months. Similarly, a meta‐analysis by Shen et al. [36] described a significantly greater visual acuity improvement using ILM‐F compared to ILM‐P at 3 months, which did not reach significance at 6 months. As visual acuity recovery is driven by the restoration of outer retinal layers, the wide range of follow‐up durations (2 months to 6.4 years) may confound unadjusted visual outcomes analyses [43]. The 3‐month timepoint in this study was chosen to most accurately reflect the typical post‐operative assessment timepoint and to minimise confounding from post‐operative cataract development.

There was a trend of more surgeons performing fewer ILM‐F procedures compared to the number of surgeons performing ILM‐P only surgeries. This may suggest less experience with the ILM‐F technique. Our data showed that despite better hole closure with consultants, visual acuity was not significantly different between consultants and trainees. However, the surgeon's experience with one technique cannot be deduced by their grade (consultant or trainee). The relative use of ILM‐F compared to ILM‐P has increased from 2017 to the end of 2023.

To our best knowledge, our study has the largest number of subjects undergoing ILM flap or ILM peeling. We are the first to systematically report macular holes exceeding 400 μm using the CLOSE study classifications, which incorporate the debated 500–600 μm cutoff point. A robust regression model was used to adjust for covariates and allowed us to test the effect of hole size and closure technique. The use of a binational multicentre registry data reduced selection bias, thereby improving the generalizability of our findings. Limitations of this study include its retrospective nature. The ILM‐F technique was not documented. Some studies have found similar closure rates between single‐layered ‘cover’ and classic ‘fill’ techniques, [40, 41, 44] whilst others have shown superior visual recovery with a pedicle transposition flap over a free flap [20]. The follow‐up of 3 months may be too short to fully appreciate visual gains following surgery. Visual acuity measurement does not account for reductions in scotoma size and metamorphopsia [45]. Other measures of vision, such as multifocal electroretinogram, microperimetry, and metamorphopsia assessments, may form a more comprehensive functional analysis.

In conclusion, the ILM‐F technique was superior to conventional ILM‐P in achieving FTMH closure but there was no additional visual acuity benefit. Given the ultimate goal of surgery is to improve visual outcomes, ILM‐F is not required in smaller and medium sized holes where MH closure rates are already high with ILM‐P. We recommend other surgical registries to study their data on ILM flaps, with particular attention to any functional benefit or detriment compared to conventional ILM peeling. More studies including comparative analyses of ILM flap techniques are required.

## Conflicts of Interest

The authors declare no conflicts of interest.

## Data Availability

The data that support the findings of this study are available from the corresponding author upon reasonable request.
